# Mutant LRRK2 exacerbates immune response and neurodegeneration in a chronic model of experimental colitis

**DOI:** 10.1007/s00401-023-02595-9

**Published:** 2023-06-08

**Authors:** Diego Cabezudo, George Tsafaras, Eva Van Acker, Chris Van den Haute, Veerle Baekelandt

**Affiliations:** 1 Laboratory for Neurobiology and Gene Therapy, Department of Neurosciences, Leuven Brain Institute, KU Leuven, Herestraat 49, box 1023, 3000 Leuven, Belgium; 2 Leuven Viral Vector Core, Herestraat 49, box 1023, 3000 Leuven, Belgium

**Keywords:** Parkinson’s disease, LRRK2, Inflammation, Inflammatory bowel disease, Alpha-synuclein

## Abstract

The link between the gut and the brain in Parkinson’s disease (PD) pathogenesis is currently a subject of intense research. Indeed, gastrointestinal dysfunction is known as an early symptom in PD and inflammatory bowel disease (IBD) has recently been recognised as a risk factor for PD. The leucine-rich repeat kinase 2 (LRRK2) is a PD- and IBD-related protein with highest expression in immune cells. In this study, we provide evidence for a central role of LRRK2 in gut inflammation and PD. The presence of the gain-of-function G2019S mutation significantly increases the disease phenotype and inflammatory response in a mouse model of experimental colitis based on chronic dextran sulphate sodium (DSS) administration. Bone marrow transplantation of wild-type cells into G2019S knock-in mice fully rescued this exacerbated response, proving the key role of mutant LRRK2 in immune cells in this experimental colitis model. Furthermore, partial pharmacological inhibition of LRRK2 kinase activity also reduced the colitis phenotype and inflammation. Moreover, chronic experimental colitis also induced neuroinflammation and infiltration of peripheral immune cells into the brain of G2019S knock-in mice. Finally, combination of experimental colitis with overexpression of α-synuclein in the substantia nigra aggravated motor deficits and dopaminergic neurodegeneration in G2019S knock-in mice. Taken together, our results link LRRK2 with the immune response in colitis and provide evidence that gut inflammation can impact brain homeostasis and contribute to neurodegeneration in PD.

**Supplementary Information:**

The online version contains supplementary material available at 10.1007/s00401-023-02595-9.

## Introduction

Parkinson’s disease (PD) is the second most common neurodegenerative disorder, affecting over 10 million people worldwide. The hallmarks of the disease are the presence of cytoplasmic protein inclusions containing mainly misfolded α-synuclein (termed Lewy Bodies and neurites), the degeneration of the dopaminergic neurons in the substantia nigra pars compacta (SNpc) and neuroinflammation. Dopaminergic dysfunction ultimately leads to the characteristic motor symptoms of PD (tremor, bradykinesia, rigidity and postural instability), but other non-motor symptoms are commonly present in PD patients. More specifically, the importance of the gut-to-brain axis in PD pathogenesis already postulated by Braak has received increasing attention recently. Indeed, constipation is present in approximately 60% of the PD patients and, as it precedes motor symptoms by decades, the research criteria of the Movement Disorder Society describe it as a predictive symptom for prodromal PD [31, 64]. In addition, inflammatory bowel disease (IBD) is a chronic condition that includes Crohn’s disease (CD) and ulcerative colitis (UC) and has been identified as a risk factor for PD [48, 60–62, 66]. Furthermore, anti-TNF-α treatment of IBD patients eliminated this increased risk for PD, supporting the relevance of gut inflammation in PD pathogenesis [50].

The leucine-rich repeat kinase 2 (LRRK2) was genetically linked to PD in 2004 with the most common mutation G2019S accounting for 4% of familial and 1% of PD sporadic cases [27]. Mutations in LRRK2 increase the kinase activity of the protein and therefore the clinical use of LRRK2 kinase inhibitors is currently being evaluated. Despite intense research in the field, the function of LRRK2 and its involvement in the pathogenesis of PD is not fully understood. A great body of evidence supports the role of LRRK2 in immune cells and inflammatory diseases (reviewed in Cabezudo et al. 2020)). Indeed, the highest expression of this protein is found in monocytes, neutrophils, dendritic cells and to a lower extent, T- and B cells [13, 14, 20, 24, 58]. Additionally, G2019S patients and asymptomatic carriers were reported to have higher levels of inflammatory cytokines [17].

Despite the high prevalence of LRRK2 mutations in familial and sporadic PD patients, the penetrance is relatively low (~ 25–40% for the G2019S mutation) [21, 36, 41]. A combination with other environmental triggers, such as inflammation, might be needed for the mutant LRRK2 to elicit its pathogenic effect. IBD, as a chronic inflammatory condition linked to PD, can potentially be such a trigger. In this context, genome wide association studies (GWAS) have associated the LRRK2 locus and IBD, and some functional variants of LRRK2 increase the risk for both CD and PD, such as the G2019S and N2081D [28, 34, 39]. However, the association between N2081D and PD is still under debate, as it is in linkage disequilibrium with the PD-linked variant rs76904798 [8, 33]. LRRK2 has been detected in immune cells from the gastrointestinal tract, and there is evidence that this expression is upregulated in antigen-presenting cells from CD patients [20]. The involvement of LRRK2 in gastrointestinal inflammation has also been suggested by some studies in rodents. An experimental colitis model in mice commonly used to study IBD and colonic inflammation is based on administration of dextran sulphate sodium (DSS). DSS was shown to increase IFN-γ and LRRK2 phosphorylation in mice, suggesting an interplay between both proteins in the gut under inflammatory conditions [53]. In addition, both LRRK2 knock-out and BAC transgenic overexpression mice demonstrated enhanced vulnerability to DSS-induced colitis [40, 56]. And recently, chronic DSS administration in a G2019S LRRK2 overexpressing transgenic mouse, induced higher gut pathology and aggravated the ongoing dopaminergic degeneration [37]. In conclusion, further research in more relevant preclinical models is needed to better understand the link between LRRK2 and colitis (Reviewed in detail in Tsafaras and Baekelandt, 2022).

In this study, we aimed to characterise in detail the role of LRRK2 in gut inflammation by modelling IBD in G2019S mutant knock-in mice. This mouse model is currently accepted to better represent the human condition than previously developed transgenic models that rely on overexpression and exogenous promoters, since the LRRK2 levels and pattern of expression remain unaltered. We found that the presence of the LRRK2 mutation significantly enhanced inflammation not only in the colon but also in the brain. We found that this response was regulated by LRRK2 specifically in the immune cells. Additionally, colonic inflammation was reduced by pharmacological inhibition of LRRK2 kinase activity. Finally, we demonstrate that the higher inflammation in the presence of the G2019S mutation impacts neuronal survival in an α-synuclein-based PD model.

## Materials and methods

### Animals and ethical approval

All animal experiments were carried out in accordance with the European Communities Council Directive of November 24, 1986 (86/609/EEC) and approved by the Bioethical Committee of the KU Leuven (Belgium) (ECD projects 051/2020 and 156/2022). For this study 8–10-week-old male C57BL/6 J (NTG, JAX 000664, RRID: IMSR_JAX:000664) and B6.Cg-Lrrk2tm1.1Hlme/J (GS LRRK2, JAX:030961, RRID:IMSR_JAX:030961) were used. Animals were housed in individually ventilated cages with free access to food and water, under a controlled light–dark cycle (12-h light–12-h dark) and temperature (21 ± 1 °C).

### Chronic DSS Scheme

DSS (molecular weight, 40 kDa, TdB Labs) was added to the drinking water at a concentration range of 0.5–2% (w/v) for 28 days. The DSS concentration was gradually increased by 0.5% w/v every 5 days and replaced with regular animal facility water for 2 days in between).

### Bone marrow transplantation

C57BL/6-Ly5.1 (B6.SJL-PtprcaPepcb/BoyCrCrl, Charles river) and B6.Cg-Lrrk2tm1.1Hlme/J were used as donors. Donor mice were euthanised by cervical dislocation without anaesthesia and the humerus, femur and tibia were used for the bone marrow (BM) isolation. Upon aseptic conditions, BM cells were isolated, filtered through a 100 μm cell strainer, washed in ice-cold PBS, and resuspended in serum free medium (RPMI, Fisher scientific). Ten-week-old recipient C57BL/6 J and B6.Cg-Lrrk2tm1.1Hlme/J mice, lethally irradiated at 9.5 Gy for 10 min, were transplanted with approximately 10 × 10^6^ cells/mouse by retro-orbital injection. Recipient mice were treated with 1 mM sulfamethoxazole and 0.18 mM trimethoprim in the drinking water for 2 weeks. Twelve weeks post transplantation the DSS administration protocol was initiated.

### MLi-2 treatment

Mice were fed with food pellets containing either MLi-2 (MedChemExpress) at a dose of 25 mg/kg of body weight per day (Research Diets, Inc.) or placebo starting 2 days prior to DSS administration and maintained for the duration of the experiment. Animals were randomly assigned to the compound or placebo groups.

### Stereotactic injections

All surgical procedures were performed using aseptic techniques and mice were treated with ketamine (60 mg/kg, i.p., Ketalar, Pfizer, Puurs) and medetomidine (0.4 mg/kg, Dormitor, Pfizer) anaesthesia. Following anaesthesia, the rodents were placed in a stereotactic head frame (Stoelting, Wood Dale, IL). Injections were performed with a 30-gauge needle and a 10-μl Hamilton syringe (Hamilton, Bonaduz, GR, Switzerland). LRRK2 GS or WT mice were injected with 2 μl rAAV2/7 αSYN vector (CMVenh-synapsin-intron-Hs αSyn WT) at a dose of 3 × 10^9^ genome copies/ml. The right SNpc was targeted using the following stereotactic coordinates: anteroposterior − 3.1 mm, mediolateral − 1.2 mm, and dorsoventral 4.3 mm (counting from the skull) using bregma as reference. The injection rate was 0.25 μl/min, with a 5-min interval following initial needle placement. The needle was left in place for an additional 5 min period after the injection procedure before being slowly retracted.

### Behavioural analysis

The motor phenotype of the rAAV2/7 αSYN vector-injected mice was assessed one month post-injection. One week prior to behavioural analysis, mice were handled on a daily basis to minimise stress and anxiety. Before each behavioural test, mice were allowed to acclimate to the testing room for a minimum of 30 min. Two behavioural tests were implemented to evaluate locomotion and forelimb asymmetry. For the rotarod, mice were placed on 5 rotating rods and were left walking face forward under steady rotation speed (4 rpm), for at least one minute (revolutions per minute). Past training time, the speed of the rotation was progressively increased from 4 to 40 rpm over 5 min. Latency to fall or passive rotation was recorded. Three trials were conducted with 1 h resting intervals in between. The mean latency for all three trials was used for analysis. For the cylinder test, mice were placed in a transparent glass cylinder and the front paw contacts to the cylinder wall were recorded as left, right, or both paws, with a total of 25 contacts per animal. The number of contralateral forelimb contacts was expressed as a percentage of total forelimb contacts.

### Flow cytometry

The animals were killed with an overdose of sodium pentobarbital (Dolethal, 200 mg/kg, i.p.), followed by intracardial perfusion with ice-cold saline. Half brains were dissected and enzymatically digested with Collagenase D (Merck Life Science BV) and DNAse I (Roche) at 37 °C, followed by passage through a 70 μm cell strainer. Cells were washed with Hank’s Balanced Salt Solution (HBSS) and centrifuged at 1000 g for 10 min. The pellet was resuspended with 38% Percoll (GE Healthcare Bio-Sciences) and centrifuged for 25 min at 800 g with slow acceleration and no brake. Cells were collected from the bottom of the tube and washed once with Dulbecco’s Modified Eagle Medium (DMEM) containing 10% foetal bovine serum (FBS) and once in FBS stain buffer (BD Biosciences).

Equal parts of distant colon samples were dissected and cleaned. After incubation in HBSS with 10% FBS and 5 mM EDTA at 37 °C, the colon pieces were washed with DMEM and minced finely. Then, each sample was enzymatically digested with Collagenase D and DNAse I at 37 °C followed by passage through a 100 μm cell strainer. Cells were washed in DMEM with 10% FBS and centrifuged at 1000 g for 10 min. The pellet was resuspended in a 40% Percoll in DMEM underlaid by a layer of 80% Percoll in DMEM and centrifuged for 25 min at 800 g with slow acceleration and no brake. The interphase was collected and washed in FBS stain buffer.

For both protocols, the cells were then blocked with mouse anti-rat FcγII receptor (CD32) (D34-485, BD Biosciences) in FBS stain buffer and stained for the following surface markers: CD45 30-F11, PE-Cy7 (Biolegend); CD11b M1/70, PerCPCy5.5- (Biolegend); Ly-6G 1A8, PE (BioLegend); CD4 GK1.5, AlexaFluor 488 (BioLegend); CD8a 53–6.7, Pacific Blue (BioLegend); CD25 PC61, PE-Cy5 (BioLegend); MHC II M5/114.15.2, APC (BioLegend); Ly-6C HK1.4, Brilliant Violet 605 (BioLegend); CD19 6D5, Brilliant Violet 570 (BioLegend); CD11c, eFluor™ 506 (ThermoFisher eBioscience^™^); CD161 PK136, Brilliant Violet 650 (BioLegend); CD68 FA-11, Brilliant Violet 711 (BioLegend); CD3 17A2, Brilliant Violet 750 (BioLegend); CD45.2 104, APC (BioLegend); CD45.1 A20, FITC (BioLegend). A fixable viability dye eFluor 780 (eBioscience) was used in each condition. Cells were fixed in 4% PFA, and data were acquired on the following day. Flow cytometry/FACS was performed at the VIB-KU Leuven FACS Core Facility using a BD FACSymphony A5 cytometer with the FACSDiva software. Data were analysed using FlowJo v10.0.7 software. Fifty thousand to one hundred thousand events were collected for each brain sample and one hundred fifty thousand events for the colon samples.

### Immunohistochemistry

Mice were killed with an overdose of sodium pentobarbital (200 mg/kg, i.p., Dolethal, Vetoquinol) followed by intracardial perfusion with saline and/or 4% PFA in PBS. After perfusion and fixation, colons were embedded in paraffin blocks, cut into 6 μm sections and mounted on glass slides. Slides were stained using standard immunohistochemistry as described below. Slides were de-paraffinised with 2 sequential 5-min washes in xylenes, followed by 5-min washes in a descending series of ethanol concentrations: 100%, 100%, 95%, 70% and finally incubated in deionised water for 5 min prior to antigen retrieval. Slides were stained for haematoxylin and eosin (H & E) staining. The histological score for the colitis was assessed based on different parameters as follows; crypts architecture: from normal morphology to loss of entire crypts morphology (0–3); presence of oedema: oedema (0–3); goblet cell depletion (0–1). The infiltration of immune cells into the lamina submucosa was scored from 0 to 3. The total histological score was calculated as the sum of the individual scores of these parameters.

For immunohistochemical analysis slides were treated with 3% hydrogen peroxide for 10 min and incubated overnight with rat anti-mouse MHC Class II (I-A/I-E) (eBioscience™) primary antibody in 10% rabbit serum (DakoCytomation) and as secondary antibody we used biotinylated anti-rabbit IgG (DakoCytomation), followed by incubation with streptavidin–horseradish peroxidase complex (DakoCytomation). MHC II immunoreactivity was visualised using 3' diaminobenzidine tetrahydrochloride (DAB; Merck Life Science BV). For immunofluorescent staining the slides were incubated overnight in PBS-0.1% triton X-100 with 10% donkey serum (Jackson ImmunoResearch Laboratories) and the following antibodies: goat Myeloperoxidase (MPO, eBioscience^™^) and rat F4/80 (Biolegend). After three rinses in PBS-0.1% triton X-100, the sections were incubated in the dark for 2 h in fluorochrome-conjugated secondary antibodies: donkey anti-goat Alexa 488 (Abcam) (MPO) and donkey anti-rat Alexa 555 (Abcam) (F4/80).

For the immunohistochemical stainings of the brain, after the post-fixation overnight in 4% PFA, 25 µm thick coronal brain sections were made with a vibrating microtome (HM 650 V, Microm). Immunohistochemistry was performed on free-floating sections using antibodies against tyrosine hydroxylase (TH, rabbit polyclonal, Merck Millipore). Sections were pretreated with 3% hydrogen peroxide for 10 min and incubated overnight with primary antibody in 10% rabbit serum (DakoCytomation) and as secondary antibody we used biotinylated anti-rabbit IgG (DakoCytomation), followed by incubation with streptavidin–horseradish peroxidase complex (DakoCytomation). TH immunoreactivity was visualised using Vector SG (SK-4700, Vector Laboratories).

### Stereological quantification of dopaminergic neurons

For the assessment of the integrity of the SNpc, the number of TH + cells was determined by stereological measurements using the optical fractionator method in a computerised system as described before [3] (Stereo Investigator; MicroBrightField, MBF). Every 8th section throughout the entire SNpc and a total of 5 sections for each animal, was analysed for TH immunoreactivity. The coefficients of error, calculated according to the procedure of Schmitz and Hof as estimates of precision, varied between 0.05 and 0.10. Percentages represent the loss of dopaminergic neurons compared to the contralateral, non-injected side. All analyses were performed by an investigator blinded to the different groups.

### Protein extraction

Colon pieces were freshly isolated, weighted and snap-frozen. Samples were homogenised in 20 volumes, respectively, of RIPA buffer (50 mM Tris–HCl, 150 mM NaCl, 0.1% (w/v) SDS, 1% (v/v) Triton-X100, 0.5% (w/v) Sodium Deoxycholate, 1.0 mM EDTA pH 7.4) containing a protease inhibitor cocktail (Roche cOmplete) and phospho-STOP EASYPACK (Roche) using a tissue homogeniser (TH, Omni Tissue Homogenizer). After 30-min incubation at 4 °C, the samples were spun for 10 min at 3000 g at 4 °C, the supernatant and was further spun for 30 min at 20.000 g at 4 °C, and the resulting supernatants represented the protein fraction. A second colon piece was homogenised in 10 volumes of urea buffer (8 M urea, 100 mM Tris–HCl, 10% glycerol, 1% SDS, 5 mM DTT, 1 mM EDTA, 1 mM EGTA, pH 6.8) containing protease and phosphatase inhibitor using a tissue homogeniser. After 30-min incubation at 4 °C, the samples were spun for 10 min at 6000 g at 4 °C, and the resulting supernatants represented the protein fraction. Protein sample concentration was determined by BCA protein assay (Thermo Scientific, MA, USA) according to the manufacturer’s directions.

### Western blot

To assess LRRK2 and LRRK2 P-S1292 by Western blot analysis, 40 μg protein of colon samples extracted in urea buffer were loaded on NuPAGE 3–8% Tris–acetate gradient gels (Invitrogen, Waltham). Separated proteins were transferred to a polyvinylidene fluoride (PVDF) membrane (Bio-Rad). Nonspecific binding sites were blocked for 15 min in PBS with 0.1% Triton X-100 (PBS-T) and 5% nonfat milk. After overnight incubation at 4 °C with primary antibodies (anti-LRRK2 N241A/34, NeuroMab; anti-LRRK2 (P-S1292), Abcam; anti-vinculin V9131, Sigma), blots were washed 3 times with PBS-T and incubated with horseradish peroxidase-conjugated secondary antibody (Dako) for 1 h and washed again 3 times. Bands were visualised using Clarity Western ECL (Bio-Rad) and developed with a GE ImageQuant 800 (GE Healthcare). Densitometric analysis was performed using ImageQuant software (GE Healthcare).

### Meso scale V-plex assay

Pro- and anti-inflammatory proteins were analysed in the protein fraction of the colon homogenates using a 96-well V-PLEX Mouse cytokine 19-plex (Proinflammatory Panel-1 and Cytokine Panel-1) (#K15255D; Meso Scale). Each mouse colon extract (homogenised in RIPA buffer) was diluted two fold with diluents and incubated for 2 h at RT. After washing with 0.05% Tween‐20 in 0.01 M PBS, the plates were incubated with detection antibodies for 2 h at RT. Data were generated from the V-PLEX Mouse cytokine 19-plex kits by washing away detection antibody solution and adding reading buffer to generate an electrochemiluminescence signal measured in a Meso Quickplex SQ120 (Meso Scale) and quantified with the Discovery Workbench 4.0 software.

### Microscopy and image analysis

H&E slides were scanned using an Aperio ScanScope CS whole slide scanner at 40X magnification. Fluorescent staining images were obtained with Leica DM6 B upright microscope using Leica Application Suite 3.6 Download. For the counting of F4/80 and MPO positive cells, tiled images of three distant colon sections/animal were counted using QuPath 0.3.2 software. For the imaging analysis, Fiji v2.0.0 software was utilised.

### Statistical analysis

Graph creation and statistical analysis were performed using GraphPad Prism for Windows (GraphPad Software, Inc.), version 9.0. Results are presented as means ± standard error of the mean. Normality of data was tested using the Shapiro–Wilk test. Data not showing normal distribution were transformed before applying the parametric ANOVA. Statistical significance was assessed using t-test or when multiple groups were analysed simultaneously, one-way ANOVA followed by Tukey’s multiple comparison post hoc test, two-way ANOVA followed by Sidak’s post hoc test, or three-way ANOVA followed by Sidak’s post hoc test. Significance was represented as follows: *p < 0.05, **p < 0.01, ***p < 0.001, and ****p < 0.0001.

## Results

### The G2019S LRRK2 mutation increases the susceptibility to DSS-induced colitis in mice

**Fig. 1:**
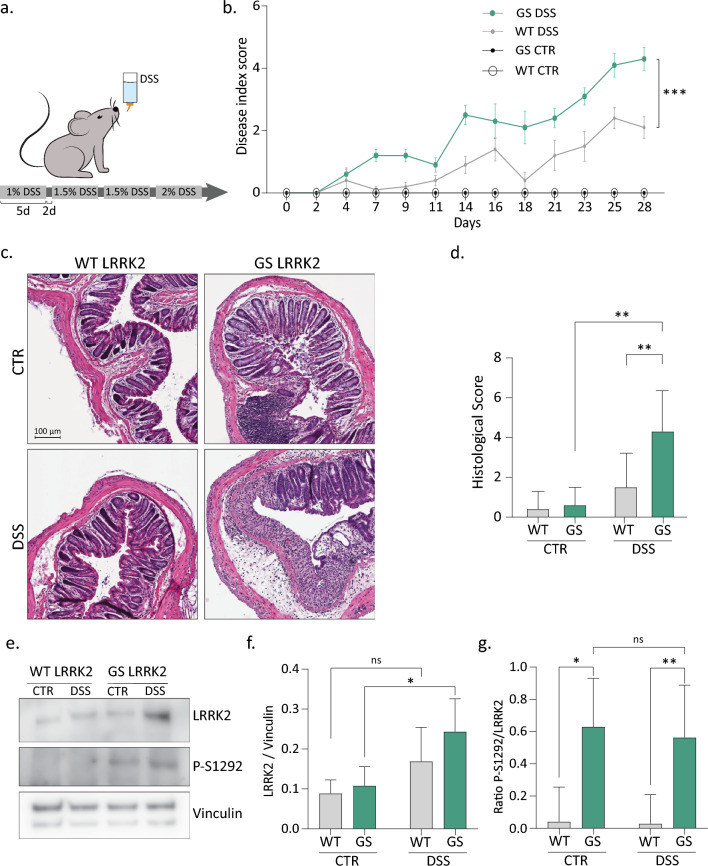
The G2019S LRRK2 mutation increases the sensitivity to experimental colitis in mice. **a** DSS administration protocol. Mice received DSS in drinking water at increasing concentrations each time for 5 days with a pause of 2 days during one month. **b** Disease index score evaluation of stool consistency and haematochezia measured three times per week revealed a more severe phenotype in GS LRRK2 mice. Graph represents mean ± SD. Statistical differences were assessed using three-way ANOVA and Tukey’s post hoc test, n = 10 animals/group. ***p < 0.001. **c** Representative image of Haematoxylin and Eosin (H&E) staining of the distal colon showing the damage to the epithelium and inflammation after DSS. Scale bar = 100 µm. **d** Histological scoring of the H&E images based on crypt architecture, infiltration of immune cells, presence of oedema and depletion of the goblet cells. **e–g** Immunoblot analysis of total and S-1292 phosphorylated LRRK2 in distal colon protein extracts of DSS-treated and control GS and WT mice. Graphs represent mean ± SD. Statistical differences were assessed using two-way ANOVA and Tukey’s post hoc test, n = 10 animals/group. *p < 0.05, **p < 0.01, ***p < 0.001

Administration of DSS in mice induces disruption of the distal colonic intestinal epithelial layer, resulting in histological damage with morphological and symptomatic resemblance to human UC. The severity and acuteness of the inflammation can be modulated by the dose and administration pattern of DSS. In this study, we first established a chronic and progressive colitis model by administration of increasing concentrations of DSS during a period of 28 days and compared the response between knock-in mice carrying the G2019S LRRK2 mutation (GS LRRK2) and control wild-type mice (WT LRRK2) (Fig. 1a). In agreement with the literature, our preliminary analysis suggested that female mice are partially protected against DSS [2, 22]. Since analysing both sexes separately was deemed necessary for a thorough investigation, we decided to exclusively utilise male mice in all subsequent experiments to ensure the appropriate statistical power.

Our DSS protocol moderately reduced the body weight but did not cause lethality, with no differences between GS LRRK2 and WT LRRK2 mice (Sup Fig. 1a). The severity of the colitis was scored three times per week by assessing the stool consistency and presence of blood in the stool (Sup Fig. 1b, c). Both parameters, as well as the combined score (disease index score), show that our chronic administration scheme induced mild experimental colitis in WT mice (Fig. 1b). Nevertheless, a significantly increased sensitivity was observed in the GS LRRK2 mice, which developed severe colitis symptoms. Additionally, the GS LRRK2 mice presented severe shrinkage of the colon, which is linked to the severity of the inflammation (Sup Fig. 1d). The histological score based on crypt architecture, infiltration of immune cells, presence of oedema and goblet cell depletion also demonstrated more damage to the colonic epithelium after DSS in the GS LRRK2 mice compared to the controls (Fig. 1c, d).

The G2019S LRRK2 mutation has been reported to increase kinase activity by approximately 4 times [65]. No difference in the basal LRRK2 protein levels was found in the colon of untreated GS and WT LRRK2 mice (Fig. 1e, f). In response to DSS, however, a significant increase in LRRK2 was found in the colon of the GS LRRK2 mice. In order to assess the kinase activity of LRRK2, we measured the autophosphorylation on Serine 1292 (Fig. 1g). Phosphorylation on S-1292 was significantly increased in GS LRRK2 mice, but to the same extent in control and DSS conditions, indicating that the kinase activity of LRRK2 remains unaltered during colitis.

**Fig. 2:**
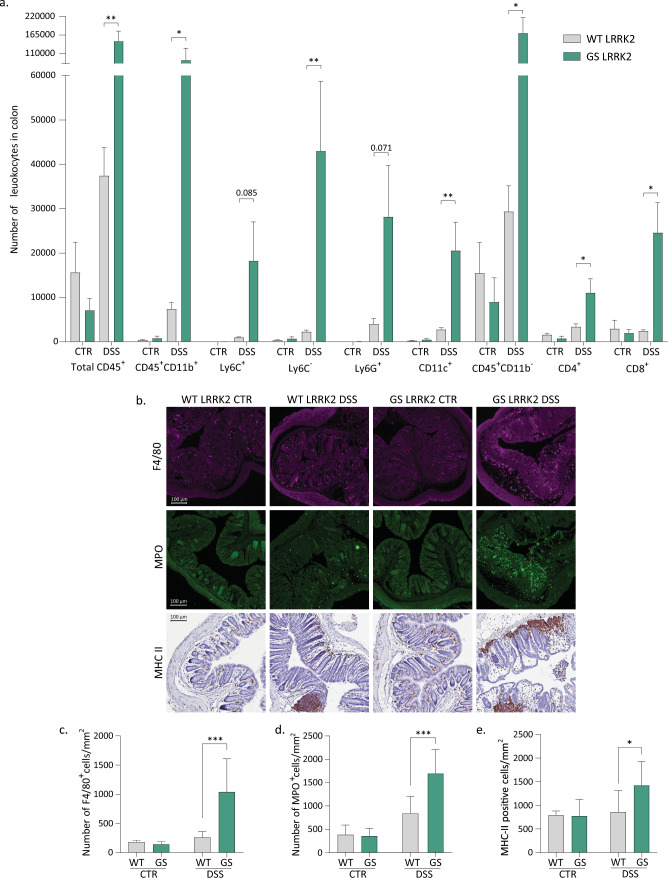
Increased inflammatory response in the colon of GS LRRK2 mice after DSS. **a** Flow cytometry immunophenotyping of the colon of WT and GS LRRK2 mice treated with DSS. Number of total leukocytes (CD45^+^), myeloid cells (CD45^+^ CD11b^+^), Ly6C^+^ and Ly6C^−^ monocytes, dendritic cells (CD11c^+^), neutrophils (Ly6G^+^), lymphocytes (CD45^+^ CD11b^−^) and T cells (CD8^+^ and CD4^+^) are shown. Graphs represent mean ± SD. Statistical differences were assessed using Student’s t-test between WT and GS DSS groups, *n* = 5 (DSS), 2 (CTR) animals/group. *p < 0.05, **p < 0.01. **b** Immunostaining of paraffin-embedded distal colon sections. Representative images of macrophage (F4/80), neutrophil (MPO) and MHC II immunostaining in the distal colon of WT and GS LRRK2 mice. Quantification of macrophages **c**, neutrophils **d**, and MHC II expressing cells. **e** Graphs represent mean ± SD. Statistical differences were assessed using two-way ANOVA and Tukey’s post hoc test, *n* = 10 animals/group. *p < 0.05, ***p < 0.001

To characterise the inflammation in the colon we first used flow cytometry immunophenotyping (Fig. 2a). Different populations of immune cells were quantified according to the gating strategy shown in the Supplementary Fig. 2. An overall increase in leukocytes (CD45^+^ cells) was found in the GS animals after DSS treatment. When looking at different subpopulations, we observed a significantly higher response in the GS LRRK2 animals treated with DSS for the non-classical (Ly6C^−^) monocytes, dendritic cells (CD11c^+^) and the lymphoid population (CD45^hi^, CD11b^−^), more specifically the CD8^+^ and CD4^+^ T cells. A similar trend that did not reach statistical significance was found for the myeloid cells (CD45^hi^, CD11b^+^), the classical (Ly6C^+^) monocytes and the granulocytes (defined as Ly6G^+^ , Ly6C^+^ , CD11b^+^ cells).

**Fig. 3:**
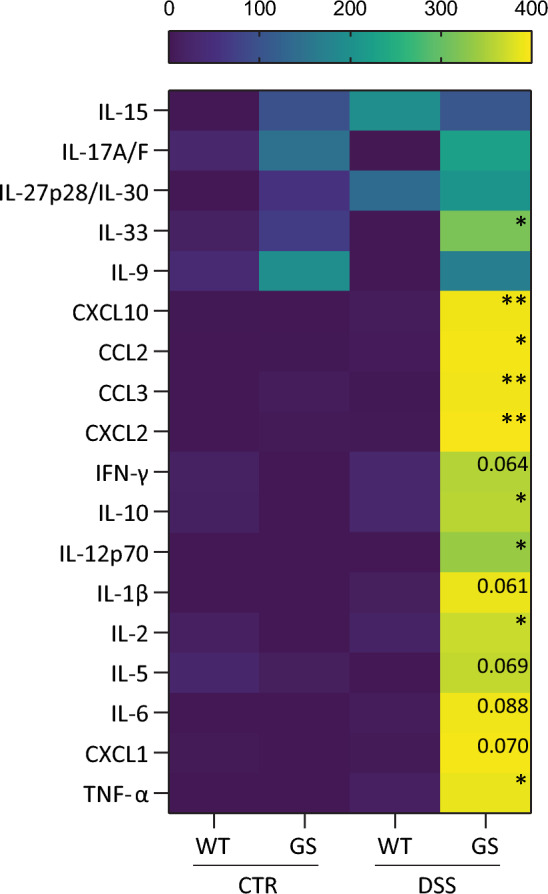
DSS induces higher levels of pro-inflammatory cytokines in the inflamed colon of GS LRKK2 mice. Cytokines were measured in colon protein extracts from WT and GS LRRK2 mice after DSS administration as well as WT and LRRK2 control mice (CTR). Increased levels of IL-33, IP-10, MCP-1, MIP-1α, MIP-2, IL-10, IL12p27, IL-2 and TNF-α, as well as trends towards increased levels of IFN-γ, IL-1β, IL-5, IL-6 and CXCL1 were found in GS mice. Graphs represent mean ± SD. Statistical differences were assessed using Student’s *t*-test between WT and GS DSS groups, *n* = 8 (DSS), 2 (CTR) animals/group. *p < 0.05, **p < 0.01

Next, we performed immunostaining in the distal colon for macrophages (F4/80), neutrophils (MPO) and for the major histocompatibility complex class II (MHC II) (Fig. 2b). In agreement with the higher level of inflammation detected by flow cytometry, the three markers were significantly increased in the GS LRRK2 mice treated with DSS (Fig. 2c, d, e). Finally, a panel of cytokines was measured in colon homogenates from these animals (Fig. 3). An overall increase in cytokine levels was present in GS LRRK2 mice after experimental colitis. More specifically, significant differences were found between WT and GS LRRK2 mice for IL-33, CXCL10, CCL2, CCL3, CXCL2, IL-10, IL-12p70, IL-2 and TNF-α, as well as a consistent trend for IFN-γ, IL-1β, IL-5, IL-6 and CXCL1.

### Bone marrow transplantation of wild type into G2019S LRRK2 mice reverts the susceptibility to DSS-induced colitis

**Fig. 4:**
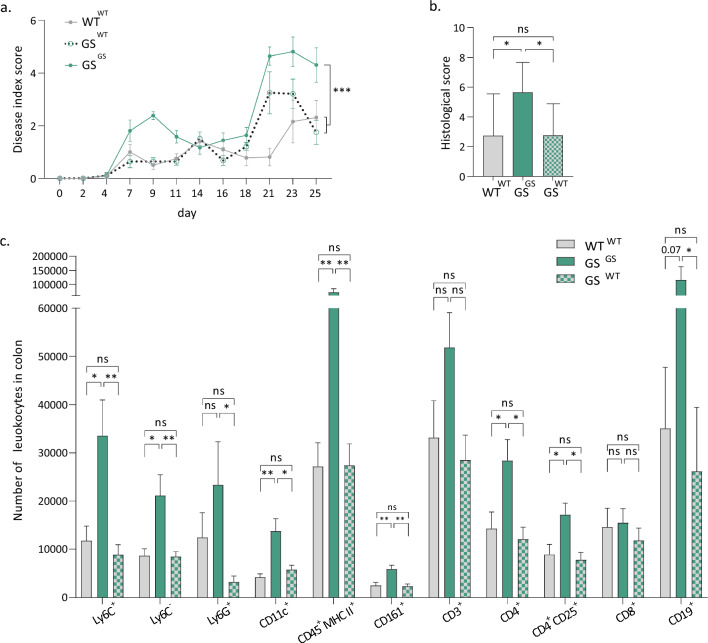
Bone marrow transplantation of WT into GS LRRK2 mice reverts the susceptibility to DSS-induced colitis. **a** Disease index score of GS mice is normalised to WT levels after bone marrow transplantation from WT mice. Graph represents mean ± SD. Statistical differences were assessed using two-way ANOVA and Tukey’s post hoc test, *n* = 8–9 animals/group. ***p < 0.001. **b** Histological score of GS mice is reduced to WT levels after bone marrow transplantation. Graph represents mean ± SD. Statistical differences were assessed using one-way ANOVA and Tukey’s post hoc test, *n* = 8–9 animals/group. *p < 0.05. **c** Colonic immunophenotyping by flow cytometry of the GS^WT^ as well as the WT^WT^ and GS^GS^ mice treated with DSS. Quantification of total myeloid cells (CD45^+^ CD11b^+^), Ly6C^+^ and Ly6C^−^ monocytes, neutrophils (Ly6G^+^), dendritic cells (CD11c^+^), MHC II^+^ antigen-presenting cells, natural killer cells (CD161^+^), lymphocytes (CD45^+^ CD11b^−^), T cells (CD3^+^), CD4 T cells, CD25^+^ CD4 T cells, CD8 T cells and B cells (CD19^+^). Graphs represent mean ± SD. Statistical differences were assessed using one-way ANOVA and Tukey’s post hoc test, *n* = 8–9 animals/group. *p < 0.05, **p < 0.01

We hypothesised that the presence of mutant LRRK2 specifically in the immune cells might be responsible for the exacerbated inflammatory response of the GS mice towards DSS. Therefore, we transplanted the bone marrow of WT mice into lethally irradiated WT (WT^WT^) and GS LRRK2 mice (GS^WT^), as well as GS bone marrow into recipient GS LRRK2 (GS^GS^) mice. To control for proper engraftment, we transplanted in parallel bone marrow from C57BL/6-Ly5.1 mice carrying the CD45.1 alloantigen of CD45 to our GS and WT LRRK2 mice which express CD45.2. 10 weeks post transplantation, engraftment was evaluated through flow cytometry (Fig. Sup. 3a). An average of 98.7% of engraftment was measured in every control animal. Next, we induced experimental colitis in the WT^WT^, GS^GS^ and GS^WT^ mice 10 weeks after bone marrow transplantation. The disease index score of the GS^WT^ group was improved compared to the GS^GS^ group and reaching similar levels as the WT^WT^ mice (Fig. 4a). The colon length was normalised in the GS^WT^ mice treated with DSS (Fig. Sup. 3b). Also, the histological score confirmed a reduction in the colonic epithelial damage in GS^WT^ mice (Fig. 4b; Fig. Sup. 3c).

Flow cytometry analysis of the colon revealed overall comparable immune cell numbers in the WT^WT^ and GS^WT^ animals in contrast to the increase seen in the GS^GS^ mice (Fig. 4c). More specifically, a rescue was found by the WT bone marrow transplantation in the GS^WT^ mice for total myeloid cells (CD45^+^ CD11b^+^), Ly6C^+^ and Ly6C^−^ monocytes, granulocytes (Ly6G^+^), dendritic cells (CD11c^+^), MHC II + leukocytes, NK cells (CD161^+^), CD4^+^ T cells, CD25^+^ expressing CD4 T cells and B cells (CD19^+^). These results demonstrate that the increased inflammation present in the GS LRRK2 mice after DSS is completely rescued by the bone marrow transplantation, suggesting that the DSS response is modulated by the activity of LRRK2 in the immune cells.

### Inhibition of LRRK2 kinase activity partially protects G2019S LRRK2 mice from DSS-colitis

To determine whether or not the increased LRRK2 kinase activity is responsible for the enhanced DSS response in the GS mice, we treated the animals with the LRRK2 inhibitor MLi-2 [19]. MLi-2 was administered using an in-diet treatment protocol as previously described for 30 days starting 2 days before the DSS treatment [49]. The dose of ingested compound was estimated at 25 mg/kg/day (mean per animal). The treatment was well tolerated and no differences in animal weight between the treatment and placebo groups were observed (Fig. Sup 4a). LRRK2 inhibition was confirmed by quantifying LRRK2 dephosphorylation at S935 in colon through western blot (Fig. Sup 4b). Our protocol results in a reduction of 80% in the phosphorylation of S935, which suggests partial inhibition of LRRK2.

**Fig. 5:**
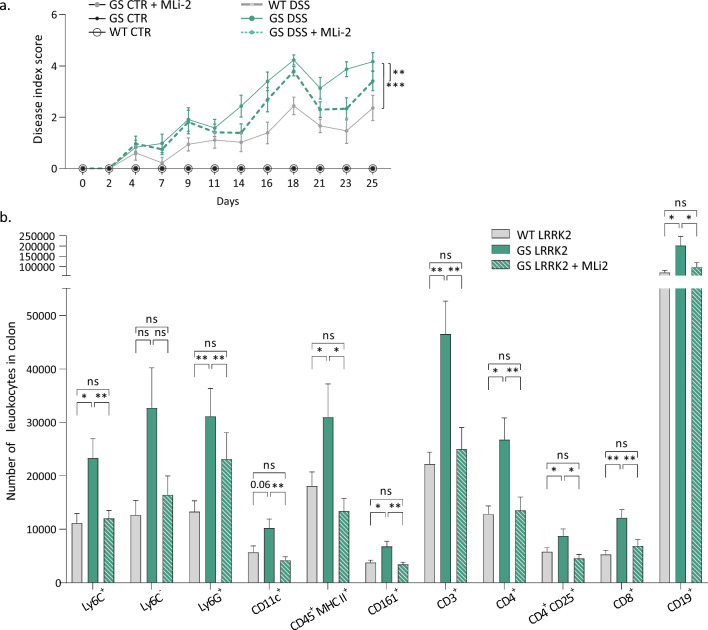
The LRRK2 kinase inhibitor MLi-2 partially rescues the enhanced susceptibility of GS LRRK2 mice to DSS. **a** Disease index score evaluation in WT and GS mice treated with placebo or MLi-2, as well as controls (CTR). Graph represents mean ± SD. Statistical differences were assessed using three-way ANOVA and Tukey’s post hoc test, *n* = 9–15 animals/group. ***p < 0.001. **b** Colonic immunophenotyping by flow cytometry of GS mice treated with MLi-2 or placebo, as well as WT mice, after DSS administration. Quantification of total number of myeloid cells (CD45^+^ CD11b^+^), Ly6C^+^ and Ly6C^−^ monocytes, neutrophils (Ly6G^+^), dendritic cells (CD11c^+^), MHC II^+^ antigen-presenting cells, natural killer cells (CD161^+^), lymphocytes (CD45^+^ CD11b^−^), T cells (CD3^+^), CD4 T cells, CD25^+^ CD4 T cells, CD8 T cells and B cells (CD19^+^). Graphs represent mean ± SD. Statistical differences were assessed using one-way ANOVA and Tukey’s post hoc test, *n* = 9–15 animals/group. *p < 0.05, **p < 0.01

Pharmacological inhibition of LRRK2 significantly ameliorated the colitis phenotype based on the disease index score, especially in the last two weeks of the treatment (Fig. 5a). Nevertheless, protection with the treatment was partial as the MLi-2-treated animals still responded more severely than the WT LRRK2 mice. The length of the colon was not significantly altered by the inhibition of LRRK2, suggesting that LRRK2 kinase inhibition at this dose and administration route could not completely counteract the GS mutation effect (Fig. Sup. 4c).

Quantification of the immune cells in the colon by flow cytometry revealed a reduction in the MLi-2 treated mice for the different immune populations tested, with the exception of Ly6C^−^ monocytes, which did not reach statistical significance (Fig. 5b). Overall, the results suggest a reduction of inflammation in the MLi-2-treated GS mice.

### Colitis aggravates dopaminergic neurodegeneration in G2019S LRRK2 mice

**Fig. 6:**
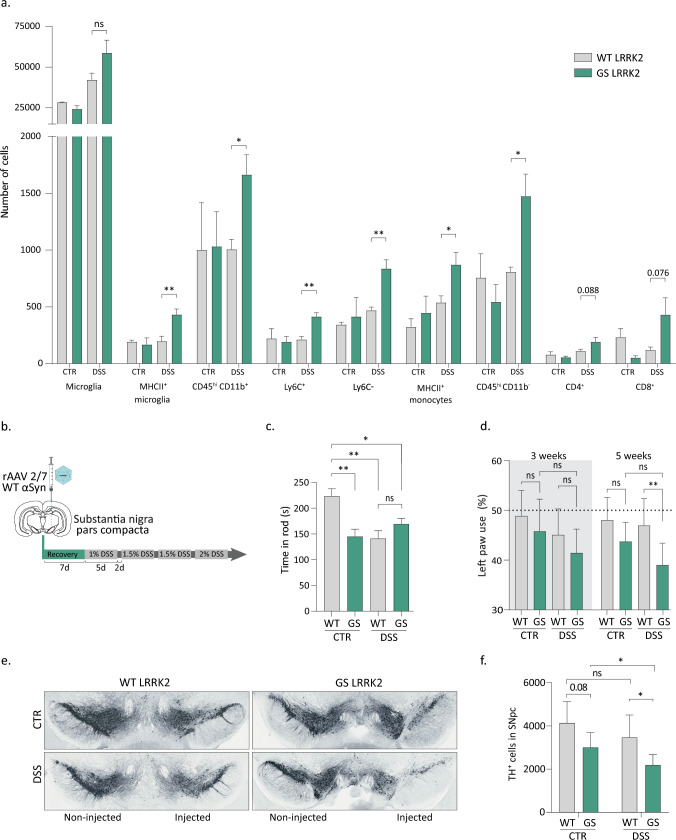
DSS-colitis aggravates α-synuclein-mediated dopaminergic neurodegeneration in GS LRRK2 mice. **a** DSS-colitis induces infiltration of immune cells in the brain of GS LRRK2 mice. Immunophenotyping of brain immune cells through flow cytometry. Graphs display number of microglia, MHC II expressing microglia, infiltrating myeloid cells (CD45^hi^ CD11b^+^), monocytes both Ly6C^+^ and Ly6C^−^, MHC II^+^ monocytes/macrophages and lymphocytes (CD45^hi^, CD11b^−^), including CD4^+^ and CD8^+^ T cells. Graphs represent mean ± SD. Statistical differences were assessed using Student’s t-test between WT and GSS DSS groups, *n* = 5 (DSS), 2 (CTR) animals/group. *p < 0.05, **p < 0.01. **b** Experimental design. Mice were unilaterally injected with α-synuclein rAAV 2/7 in the substantia nigra. DSS was administered starting 7 days after surgery. **c** Rotarod test. Graph displays the time to fall from the rotarod 5 weeks post-injection. **d** Cylinder test. Graph displays the % left forepaw use 3 and 5 weeks after α-synuclein rAAV injection in WT and GS mice with or without DSS (CTR). **e** TH immunostaining on coronal sections of the SN 5 weeks after α-synuclein rAAV injection in WT and GS mice with or without DSS (CTR). **f** Stereological quantification of TH + cells in the right SN 5 weeks p.i. Graphs represent mean ± SD. Statistical differences were assessed using two-way ANOVA and Tukey’s post hoc test, *n* = 8–10 animals/group. *p < 0.05, **p < 0.01

Inflammatory bowel disease has been reported to increase the risk for developing PD by 20–90% [48, 54, 60]. Furthermore, anti-inflammatory treatment of IBD patients with anti-TNF-α significantly reduces this risk [50]. Despite being protected by the blood–brain barrier, communication between the brain and the peripheral organs has been described. Inflammation in the gastrointestinal tract can have an effect on the central nervous system (CNS) homeostasis and induce neuroinflammation [26, 57]. To study the neuroinflammatory status after experimental colitis, we performed flow cytometry of the brain (Fig. 6a; Fig. Sup. 5). Following our chronic DSS administration protocol, modest neuroinflammation was observed in the GS LRRK2 but not WT mice. While no significant differences were found in the total number of microglial cells (CD45^med^, CD11b^+^), a higher number of microglia expressing MHC II were detected in the GS animals compared to the WT mice. This microglial response was accompanied by the infiltration of peripheral immune cells. Indeed, the GS mice presented a higher number of total myeloid cells (CD45^hi^, CD11b^+^) including both Ly6C^+^, Ly6C^−^ and MHC II^+^ monocytes. In addition, the infiltration of lymphocytes (CD45^hi^, CD11b^−^) was also increased in the GS mice, with similar trends found for both CD4^+^ and CD8^+^ T cells. We hypothesise that LRRK2 might be a key player in this gut-to-brain communication. Thus, we decided to explore whether DSS-induced inflammation could potentially compromise neuronal health. First, we checked dopaminergic cell viability after chronic DSS treatment and found no differences in TH^+^ cell counts in WT or GS mice (Fig. Sup. 6). Since dopaminergic neurodegeneration might require a combination of deleterious triggers, we decided to combine the DSS-colitis with our in-house PD mouse model based on human α-synuclein overexpression in the substantia nigra using recombinant adeno-associated vectors (rAAV 2/7). This model displays progressive dopaminergic neurodegeneration, behavioural deficits and neuroinflammation [46]. Experimental colitis was induced using the chronic DSS administration protocol starting 7 days after vector injection (Fig. 6b). The disease index score confirmed the significantly stronger phenotype in the GS LRRK2 mice (Fig. Sup.7).

Motor behaviour was examined using different tests. Rotarod test was used to evaluate the muscular coordination and grip strength of the animals 5 weeks after rAAV injection (Fig. 6c). Remarkably, a significant decrease in motor performance was found in the GS injected animals compared to the WT controls in the absence of DSS. DSS increased the motor phenotype in the WT mice, but no additional effect was observed in the GS mice. In parallel, the cylinder test was used to measure the forelimb asymmetry at 3 and 5 weeks post-injection (Fig. 6d). We observed a progressive reduction in the use of the contralateral left paw which only reached significance at the last time point between the WT and GS mice injected with α-synuclein rAAV combined with DSS, suggesting a synergistic effect between the α-synuclein overexpression and the colon inflammation.

Stereological quantification of the dopaminergic neurons in the substantia nigra corroborated the behavioural data (Fig. 6e, f). A trend towards increased neurodegeneration was observed in the GS LRRK2 mice after α-synuclein overexpression. Furthermore, DSS significantly aggravated this TH^+^ cell loss in the GS LRRK2 mice but not in the WT control mice. These results confirm that in GS LRRK2 mice, DSS-experimental colitis can aggravate α-synuclein-mediated dopaminergic neurodegeneration.

## Discussion

For decades, PD has been considered a disease strictly affecting the CNS. A growing body of evidence is now supporting an interplay between the gastrointestinal tract and the brain (gut–brain axis) [23]. Importantly, pathological hallmarks of PD, such as α-synuclein accumulation, have been found in the gastrointestinal tract prior to PD diagnosis [4, 6, 51]. Additionally, different studies have shown that inflammatory diseases of the gastrointestinal tract such as CD and UC are associated with 20–90% higher risk of developing PD and this risk is reduced again after anti-TNF-α treatment [38, 48, 50, 60, 62]. GWAS have discovered common genetic links between these diseases [30]. The most common genetic factor in familial PD, LRRK2, has been recently linked with IBD, with the mutations G2019S, associated with 4% of familial and 1% of sporadic PD cases, and N2081D described as risk factors for CD [34, 39, 52]. Therefore, LRRK2 might be involved in the connection between PD and IBD.

In this study, we aimed to investigate the role of LRRK2 in gastrointestinal pathophysiology and the potential effect in the CNS. Hence, we modelled UC using DSS in G2019S LRRK2 knock-in mice and show a more severe disease phenotype and an increased immunological response. To date, a few studies have already tried to evaluate the role of LRRK2 in colitis in different rodent models with divergent results. Indeed, WT and G2019S LRRK2 BAC transgenic mice were found to be more sensitive to acute DSS administration, involving LRRK2 in the activation of the NFκB signalling cascade [37, 56]. On the other hand, a dampened immune response was shown in G2019S LRRK2 overexpression rats [47]. In LRRK2 knock-out animals, both a more severe and a milder phenotype have been described [40, 56]. Here, we opted to use G2019S LRRK2 knock-in mice for the first time [65]. In these mice, the G2019S mutation has been introduced in the WT LRRK2 gene resulting in unaltered physiological expression pattern and protein levels. We observed a significantly more severe response to chronic DSS in the GS LRRK2 mice. Histological analysis of the distal colon not only revealed a higher damage in the epithelium, but also a robust infiltration of immune cells into the submucosal layer and lamina propria. DSS acts as a physical agent that disrupts the epithelial cell barrier of the colon, causing the normal mucosal microbiome to activate the immune response. Previous studies with this model have shown that the first responders are DC, monocytes/macrophages, and neutrophils, which have also been found in IBD patients [5, 7, 55]. This is followed by activation of the adaptive immune system through lymphocytes [25]. We report an increase in the number of DC, monocytes and a trend toward increased neutrophils in the lamina propria in our colitis model. Higher activation of the myeloid cells after chronic DSS in the GS LRRK2 mice is in line with the reported higher expression of LRRK2 in the myeloid cells than in the T and B cells [13, 14, 20, 24, 58]. Previous studies have reported that G2019S LRRK2 modifies the chemotactic response of myeloid cells by modulating the cytoskeleton through actin. An increased response to different chemoattractants, including LPS, ADP, and a model of thioglycolate-induced peritonitis, was observed [42]. In addition, fibroblasts from mice or patients with LRRK2 mutations have increased motility and actin dynamics, which can promote cell motility and chemotaxis [10, 11]. Furthermore, inhibition of LRRK2 kinase has been shown to attenuate chemotaxis to α-synuclein fibrils in primary cultured macrophages, as well as the infiltration of peripheral monocytes into the CNS [63]. We also observed an enhanced infiltration of both CD4 and CD8 T cells, which could be directly linked to the hyperactivity of G2019S LRRK2 or secondary to the stronger activation of the antigen-presenting cells (APCs), such as DC and monocytes/macrophages. Indeed, a robust increase in MHC II expression was found in the GS LRRK2 mice, indicative of an activation of the APCs.

Cytokines play a key role in the control of mucosal inflammation in IBD. Specific patterns of cytokine production have been described in immune cells from the periphery and lamina propria in IBD patients [44]. In this study, we measured several cytokines in the distal colon of the GS LRRK2 and WT mice. Interestingly, with our chronic DSS protocol high levels of cytokines were induced only in the GS LRRK2 mice, while WT animals remained at low levels of inflammation. Under inflammatory conditions, the DCs and macrophages from the lamina propria produce large amounts of pro-inflammatory cytokines such as IL-1b, IL-6, IL-18 and TNF-α [45]. IL-12 is also highly produced by intestinal myeloid cells in CD [43]. In line with the cytokines described in IBD, we found an increase in IL-6, TNF-α and IL-12 and a trend for higher IL-1b. The anti-inflammatory cytokine IL-10, as well as the pro-inflammatory cytokine IFN-γ, mostly produced by T cells, were also increased in the inflamed colon of our GS mice. Of interest, IFN-γ knock-out mice do not develop colitis after DSS, showing the importance of this cytokine in experimental colitis [29]. On the other hand, IFN-γ levels were reported moderately increased in chronic but not acute DSS models [35]. In addition, LRRK2 expression is upregulated by IFN-γ levels in CD [20]. Together with the higher number of immune cells present in our GS mice, this might explain the increased LRRK2 protein levels that we observed in the colon after DSS administration. We also found a modest but significant increase in IL-33 levels in our colitis model. Although this cytokine is increased in IBD patients, it is unclear whether it plays a protective or detrimental role [12]. In agreement with these studies, we detected a robust increase in the chemokines CXCL10, CCL2, CCL3, CXCL2 and CXCL1 which correlates with the recruitment of immune cells into the inflamed tissue. These chemokines also play an important role in angiogenesis, which is of particular interest for IBD, as this process is essential for leukocyte migration and repair of the damaged tissue [1].

Overall, the immune cell infiltration and cytokine profile measured in our model indicate considerable similarities with the immune response observed in IBD patients. Since only the GS mice responded severely to the inflammatory insult at the DSS dose used in this study, we hypothesised that the LRRK2 mutation in the immune cells primarily drives the increased susceptibility to DSS. The bone marrow transplantation experiment confirmed our hypothesis, since substitution of the GS LRRK2 mice immune cells with WT cells could rescue the colitis disease phenotype and reduce the inflammation in the colon.

Several LRRK2 kinase have been developed as a possible therapeutic approach for PD [18]. MLi-2, one of the most potent and blood–brain barrier permeable inhibitors, has already been tested in many in vitro and in vivo PD studies [19, 49]. In vivo, acute or subchronic administration of MLi-2 potently inhibits LRRK2 kinase activity, as measured by dephosphorylation of LRRK2 pSer935 LRRK2, both in the CNS and in peripheral tissues [19]. In this study, oral administration of MLi-2 in G2019S LRRK2 mice starting prior to DSS administration partially rescued the disease phenotype with an overall reduction of intestinal inflammation to the level of the WT mice. The dose and administration scheme of MLi-2 applied here resulted in an average of 80% reduction in P-S935 LRRK2 phosphorylation as measured by western blot. While dephosphorylation of S935 is not a direct measurement of LRRK2 kinase activity, this marker has been correlated with MLi-2 enzyme occupancy and is commonly used to estimate LRRK2 kinase inhibition [16, 19, 49]. Although a higher inhibition of LRRK2 might be necessary to achieve full rescue, our results indicate that pharmacological inhibition of LRRK2 can be protective against colitis.

Next to the robust inflammation in the colon, the chronic DSS-experimental colitis protocol also triggered neuroinflammatory events in the GS LRRK2 mice. This suggests that the G2019S LRRK2 mutation exacerbates the immune response facilitating the infiltration of immune cells into the brain and leading to neuroinflammation. Although no significant increase in microglial number could be detected, an MHCII upregulation was detected suggesting a microglia response. At the same time, a higher number of infiltrating monocytes and lymphocytes was observed, indicating high influx of peripheral immune cells as a result of a disrupted blood–brain barrier. This generated the question whether these neuroinflammatory events could provide a solid explanation for the missing link between IBD and PD. Inflammation in the CNS is currently considered a hallmark of PD, which can facilitate a neurotoxic environment for dopaminergic neurons and/or provide the prerequisites for the pathological manifestation of α-synuclein. Neuroinflammation induced by gastrointestinal inflammation has been previously described in animal models [15, 26, 57]. It was also shown that mutant LRRK2 can facilitate the crosstalk between immune responses in the periphery and the brain, leading to neurodegeneration [32]. However, in our experimental colitis model we did not detect loss of the dopaminergic neurons in the SN in the WT mice or in the GS LRRK2 mice, despite the observed neuroinflammatory changes. Therefore, we combined DSS administration with viral vector-mediated overexpression of α-synuclein in the substantia nigra [46]. In this double-hit model, we observed a worsened motor performance and enhanced neurodegeneration in the SNpc of GS LRRK2 mice, confirming the hypothesis that DSS-induced neuroinflammation can aggravate α-synuclein-induced neurodegeneration in the presence of the mutation. These data are in line with a recent study in old G2019S LRRK2 overexpressing mice, which develop spontaneous neurodegeneration with ageing, and where multiple cycles of DSS administration accelerated this neurodegenerative process [37].

## Conclusions

In this study, we show that LRRK2 plays a crucial role in colitis, through the modulation of the immune response. The presence of the hyperactive G2019S LRRK2 mutation enhances the inflammatory response in experimental colitis and, remarkably, induces neuroinflammatory events in the brain confirming the gut-to-brain crosstalk. Using bone marrow chimeric mice we prove, for the first time, that the mechanistic involvement of LRRK2 is immune cell-mediated. Importantly, our results show that pharmacological inhibition of LRRK2 kinase activity rescues the exacerbated colitis phenotype. This not only confirms the kinase-mediated effect but also suggests that LRRK2 inhibitors can be a novel and potential therapeutic intervention for LRRK2 IBD patients. Next, to shed light into the link between IBD and PD we induced colitis in a LRRK2-PD mouse model. Our data indicate that upon colitis mutant LRRK2 aggravate neurodegeneration, through a gut-to-brain-induced neuroinflammation. In conclusion, we provide experimental evidence that intestinal inflammation can significantly impact CNS homeostasis and that LRRK2 plays an important role in this process. The cellular and molecular pathways and the specific role of LRRK2 in this crosstalk between inflammatory diseases and PD still need to be further clarified. This might pave the way for new therapeutic avenues for PD and IBD.

## Supplementary Information

Below is the link to the electronic supplementary material.

Supplementary file1 Figure 1 Detailed phenotypic characterisation of the DSS response in GS LRRK2 mice (a) Average changes in body weight relative to baseline of DSS-treated vs control GS and WT mice, throughout the DSS administration period. The data represent the average weight of each animal compared to the baseline weight at the beginning of the protocol. (b, c) Disease index score was evaluated based on stool consistency and haematochezia. Consecutive changes in stool consistency (b) and haematochezia (c) were monitored for each animal and the average score of each group is presented. (d) Quantification of colon length of WT and GS groups with and without DSS treatment. Graphs represent mean ± SD. Statistical differences were assessed using three-way ANOVA and Tukey’s post hoc test, n = 10 animals/group. *p < 0.05, **p < 0.01, ***p < 0.001 (EPS 1125 KB)

Supplementary file2 Figure 2 Flow cytometry gating strategy used for colon samples. Lymphocytes were gated based on SSC-A versus FSC-A and singlets were selected from the FSC-A versus FSC-H dot plot, while the dead cells were excluded with Fixable Viability Dye. The defined populations were: Lymphocyte subpopulation (CD45high CD11b-) including total CD4 + T cells (CD4 + CD3 +), CD25+ CD4 + T cells (CD25+ CD4 + CD3 +), CD8 + T cells (CD8 + CD3 +), B cells (CD19+ MHC II + CD3-) and NK cells (CD161 + CD3 −). The myeloid lineage was further categorised in Neutrophils (Ly6C+ Ly6G+), Ly6C- Monocytes (Ly6C- Ly6G-), Ly6C- Monocytes (Ly6C- Ly6G-), Dendritic cells (CD11c+) and total MHC II+ Myeloid cells (MHC II+) (EPS 4275 KB)

Supplementary file3 Figure 3 Bone marrow transplantation of WT immune cells into GS mice. (a) Flow cytometry analysis confirms the successful engraftment of WT (CD45.1+) bone marrow cells to GS (CD45.2+). As control groups WT (CD45.2+) mice transplanted with bone marrow from WT (CD45.1+) and GS (CD45.2+) mice transplanted with bone marrow from GS (CD45.2+) are visualised. (b) Quantification of colon length of WTWT, GSGS, and GSWT groups with chronic colitis. Graphs represent mean ± SD. Statistical differences were assessed using one-way ANOVA and Tukey’s post hoc test, n = 8–9 animals/group. *p < 0.05. (c) Representative image of Haematoxylin and Eosin (H&E) staining of the distal colon showing the damage to the epithelium and inflammation after BMT-DSS (EPS 56121 KB)

Supplementary file4 Figure 4 Phenotypic characterisation of the DSS response in GS LRRK2 mice treated with the LRRK2 kinase inhibitor Mli-2. (a) Average changes in body weight relative to the baseline of Mli-2-treated vs control GS mice after DSS administration. (b) Western blot images from colon lysate of animals treated with Placebo or MLi-2 diet and assayed for pS935 LRRK2, total LRRK2 and Vinculin levels. Quantification of ratio of the P-S935 to the total LRRK2 levels of all diet-treated animals normalised to the Placebo. ***p < 0.001. (c) Quantification of colon length of WT and GS groups with and without Mli-2 after DSS administration. **p < 0.01 (EPS 1896 KB)

Supplementary file5 Figure 5 Flow cytometry gating strategy used for brain samples. Lymphocytes were gated based on SSC-A versus FSC-A and singlets were selected from the FSC-A versus FSC-H dot plot, while the dead cells were excluded with Fixable Viability Dye. The defined populations were: Lymphocyte subpopulation (CD45high CD11b-) including total CD4 + T cells (CD4 + CD3 +), CD8 + T cells (CD8 + CD3 +). The myeloid lineage was further categorised in Ly6C- Monocytes (Ly6C- Ly6G-) and Ly6C- Monocytes (Ly6C- Ly6G-). The microglia were defined as DC45medium CD11b+, which was further gated for MHC II+ Myeloid cells (MHC II+) (EPS 3504 KB)

Supplementary file6 Figure 6 Chronic DSS-colitis administration does not induce dopaminergic cell loss in the SN of WT and GS mice. Stereological quantification of the TH+ cells in the substantia nigra of WT and GS mice with or without colitis. Graphs represent mean ± SD. Statistical differences were assessed using two-way ANOVA and Tukey’s post hoc test, n = 10 animals/group (EPS 670 KB)

Supplementary file7 Figure 7 Disease index score after DSS in WT and GS mice unilaterally injected with α-synuclein rAAV 2/7 in the substantia nigra. The score was evaluated three times per week and confirmed the more severe phenotype in the mice carrying the GS mutation. Graph represents mean ± SD. Statistical differences were assessed using two-way ANOVA and Tukey’s post hoc test, n = 8–10 animals/group. ***p < 0.001 (EPS 712 KB)

## Data Availability

All data generated or analysed during this study are included in this published article [and its supplementary information files].
